# Effect of Acupuncture at LR3 on Cerebral Glucose Metabolism in a Rat Model of Hypertension: A ^18^F-FDG-PET Study

**DOI:** 10.1155/2018/5712857

**Published:** 2018-04-26

**Authors:** Jing Li, Yumei Wang, Kejie He, Chong Peng, Peilong Wu, Chenyun Li, Xinsheng Lai

**Affiliations:** ^1^Clinical School of Acupuncture and Rehabilitation, Guangzhou University of Chinese Medicine, Guangzhou, Guangdong 510405, China; ^2^First Clinical College, Guangzhou University of Chinese Medicine, Guangzhou, Guangdong 510405, China

## Abstract

Our objective was to investigate the effect of acupuncture at LR3 on cerebral glucose metabolism in spontaneously hypertensive rats (SHRs). We used ^18^F-2-fluoro-deoxy-D-glucose positron emission tomography (^18^F-FDG-PET) to examine the effects of acupuncture at LR3 on cerebral glucose metabolism in SHRs. SHRs were randomly allocated to receive no treatment (SHR group), needling at LR3 (SHR + LR3 group), or sham needling (SHR + sham group). Rats received 10 min acupuncture once per day for 7 days and were compared to normotensive Wistar Kyoto (WKY) rats. Blood pressure (BP) measurement and PET were performed after the first needling and the 7-day treatment period. BP was lower in the SHR + LR3 group compared to the other SHR groups between 30 and 60 min after the first needling and at 24 and 48 h after the 7-day treatment period. Glucose metabolism in the motor, sensory, and visual cortices was decreased in SHR group compared to WKY group. Needling at LR3 was associated with decreased glucose metabolism in the dorsal thalamus, thalamus, and hypothalamus and with increased metabolism in the cerebellar anterior and posterior lobes, medulla oblongata, and sensory cortex compared to the SHR group. These findings suggest that LR3 acupuncture improves hypertension through a mechanism involving altered brain activation in SHRs.

## 1. Introduction

Hypertension is characterised by a sustained increase in arterial BP and is usually associated with heart disease, peripheral vascular disease, and stroke [1]. Pharmacological therapy for hypertension has some disadvantages, such as costs and adverse effects [2].

Acupuncture is a complementary approach to BP management. It is an ancient treatment technique in traditional Chinese medicine which has been used for more than 3000 years [3, 4]. In recent years, many randomized controlled trials have demonstrated that needling at LR3 regulates essential hypertension in patients [5–7], and animal experiments have also indicated that moderate stimulation of LR3 has a significant effect on BP of SHRs [8–11]. Additionally, our previous study found that needling at LR3 altered the expression of six proteins in the medulla of SHRs [12]. A fMRI study found that acupuncture regulated the cardiovascular system through a neural network involving the hypothalamus, cortex, and brainstem [13]. Yet, the exact mechanisms underlying effects of acupuncture on BP remain unclear.

Positron emission tomography-computer tomography (PET-CT) is an advanced brain functional imaging technique that is used to measure regional glucose metabolism as an indicator of brain activity. PET-CT is characterised by high sensitivity, improved resolution, and the ability to provide semiquantitative data compared to fMRI [14, 15]. Acupuncture stimulation of specific acupoints such as LR3 affects the cardiovascular system and accordingly may have effects on specific brain regions receiving input from somatic afferent stimulation to affect BP [8, 10, 16]. Therefore, we hypothesised that acupuncture at LR3 would alter cerebral glucose metabolism as measured by PET-CT in a manner associated with lowered BP.

## 2. Methods

### 2.1. Experimental Animals

Forty-eight 10-week-old spontaneous hypertensive rats (SHRs) and 12 WKY rats weighing 200–250 g were provided by Beijing Vital River Laboratory Animal Technology Co., Ltd. (Beijing, China). The rats were maintained in an environment with controlled temperature (20–24°C) and 40–60% relative humidity on a 12-hour light/dark cycle (lights on at 07:00) at the Experimental Animal Centre of Guangzhou University of Chinese Medicine in Guangzhou, China. Rats had free access to standard diet and distilled water. All experimental procedures were conducted in accordance with the People's Republic of China Ministry of Science and Technology Laboratory Animal Care and Use Guidelines and were approved by the Committee on the Ethical Use of Animals.

### 2.2. Grouping and Acupuncture Treatment

Healthy WKY rats were used as normotensive controls (WKY group, *n* = 12). SHRs were randomly divided into 3 groups: an untreated group (SHR group, *n* = 12), a group treated with acupuncture needling at LR3 (SHR + LR3 group, *n* = 12), and a sham acupuncture group (SHR + sham group, *n* = 12). The SHR + LR3 group received acupuncture at bilateral LR3 located on the back of the foot. We selected the position of the sham point as between the 3rd and 4th toes on the back of the foot (a nonacupoint) for the SHR + sham group. Briefly, acupuncture needles (stainless steel; 13 mm in length, 0.25 mm in diameter; SUIXIN, Suzhou Hualun Medical Appliance Co., Ltd., China) were inserted at LR3 and twirled at a frequency of 90 ± 5 rotations/min with an angle of 120 ± 5°. Each treatment lasted for 10 min (5 min per side) and rats were treated once per day for 7 days. The same acupuncturist performed all acupuncture and sham treatments.

### 2.3. BP Measurement

The Kent Scientific CODA noninvasive BP measurement system was used to screen rats. Each measurement had 15 cycles and the average BP value was used. The standard for hypertension was SBP ≥ 140 mmHg or diastolic blood pressure (DBP) ≥ 90 mmHg. SHRs that did not meet the high criteria were excluded from the experiment. BP was measured between 9:30 am and 2:30 pm when it was stable. BP was measured before treatment, at 30, 60, and 90 min after acupuncture, and at 24, 48, and 72 h after the 7-day treatment period.

### 2.4. ^18^FDG-PET Imaging

All imaging was performed at the animal molecular imaging platform of Sun Yat-sen Medical College. Cerebral PET images were acquired 60 min after acupuncture and 24 h after the 7-day treatment period. Blood glucose values were measured from tail blood after 24 h of fasting using the glucose oxidase method (Glucose Kit, bioMérieux, normal standard 3.95 ± 1.31 mmol/L) prior to PET. A tracer (fludeoxyglucose [^18^F]) ^18^F-FDG synthesised with the Mini Tracer accelerator was injected (1.5 mci/kg) intravenously via the tail vein 45 min after acupuncture treatment and scanned within 15 min. Rats were anesthetised with 5% isoflurane in 100% oxygen 5 min before scanning. We used a Siemens Inveon PET system (Siemens Medical Solutions) with a radial spatial resolution of 1.4 mm full-width at half-maximum at the centre of the field of view to capture FDG-PET images. We then reconstructed images on the 128128159 matrix using the filtered backprojection algorithm, where the voxel size is equal to 1.461.460.79. All scans were saved in the Analyze 7.5 format.

### 2.5. Statistical Analysis

Data are presented as the mean ± SD. A one-way analysis of variance was used to evaluate between-group differences in BP. Analyses were performed with SPSS version 17.0 and the threshold for statistical significance was *p* < 0.05.

PET images were analysed using spmratIHEP in the SPM8 toolbox. Images were preprocessed with spatial normalisation and smoothing. Two-sample *t*-tests were used to identify differences in FDG signal between two groups. Brain regions demonstrating significant between-group differences in FDG were identified based on a voxel-level height threshold of *p* < 0.001 (uncorrected) and a cluster-extent threshold of 20 voxels.

## 3. Results

**Figure 1:**
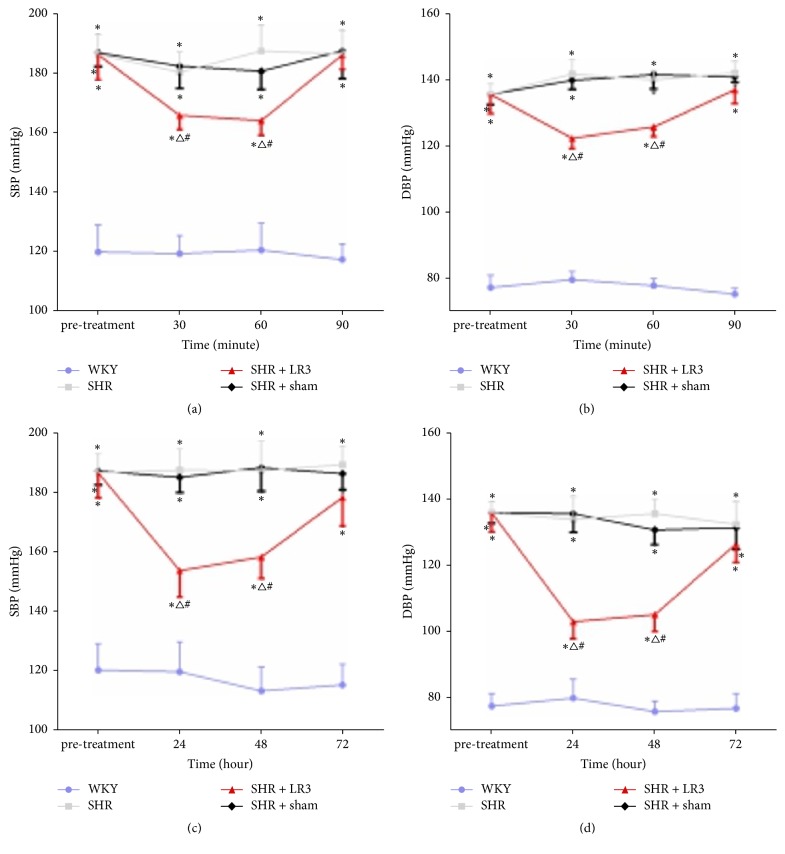
Effects of needling at LR3 in spontaneous hypertension rats (SHR). Between-group differences in systolic blood pressure (SBP; (a) and (c)) and diastolic blood pressure (DBP; (b) and (d)) were detected at 30, 60, and 90 min after initial acupuncture and at 24, 48, and 72 h after completion of the 7-day treatment period. ^*∗*^*p* < 0.05 versus the WKY group; ^△^*p* < 0.05 versus the SHR group; ^#^*p* < 0.05 versus the SHR + sham group.

Before treatment, SBP and DBP were higher in the SHR groups than in the WKY group (*p* < 0.05) (Figure 1). Compared to sham or no treatment, acupuncture at LR3 reduced elevated SBP and DBP values in SHRs from 30 to 60 min after initial needling (*p* < 0.05) (Figures 1(a) and 1(b)). SBP and DBP values remained lower in the SHR + LR3 group compared to the SHR and SHR + sham groups at 24 and 48 h after completion of the 7-day treatment period (*p* < 0.05) (Figures 1(c) and 1(d)).

### 3.1. Brain Regional Glucose Metabolism

**Figure 2:**
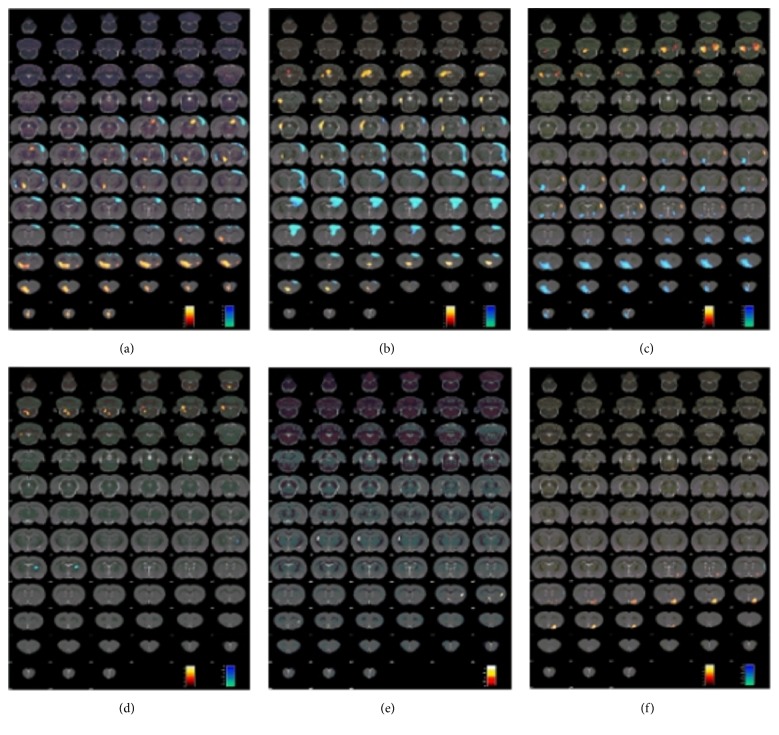
Regional glucose metabolism in the rat brain. Regional glucose metabolism was scanned at 60 min after initial acupuncture ((a), (c), and (e)) or 24 h after completion of the 7-day treatment period ((b), (d), and (f)). Results are overlaid on a coronal view of the rat brain and mapped to the Paxinos and Watson rat brain atlas. ((a) and (b)) SHR group versus WKY group. ((c) and (d)) SHR + LR3 group versus SHR group. ((e) and (f)) SHR + LR3 group versus SHR + sham group. Colour bars represent the *t*-value of each significant voxel.

Tables 1–3 summarise the brain regions with significant differences in glucose metabolism between the SHR and WKY groups, SHR + LR3 and SHR groups, and SHR + LR3 and SHR + sham groups. Significant differences in maximum *t*-values (Max_*T*) in each cluster reflect positions as per Paxinos and Watson space maximum effects. The *x*-axis from left to right is negative to positive, the *y*-axis from low to high is dorsal to ventral, and the *z*-axis from negative to positive is direction from the cerebellum to the olfactory bulb. Using the Paxinos and Watson atlas, brain regions with significant differences in glucose activity were fused on structural sections of the rat brain (Figure 2). The warm colour represents increased glucose metabolism, whereas the cold colour reflects decreased glucose metabolism.

On days 1 and 8, glucose metabolism was higher in the SHR group than in the WKY group in the thalamus, dorsal thalamus, hypothalamus, and orbital cortex. Glucose metabolism was lower in the SHR group than in the WKY group in the motor cortex, sensory cortex, and visual cortex (Table 1 and Figures 2(a) and 2(b)).

On days 1 and 8, glucose metabolism was higher in the SHR + LR3 group than in the SHR group in the cerebellum anterior lobe, cerebellum posterior lobe, medulla oblongata, and sensory cortex. Glucose metabolism was lower in the SHR + LR3 group than in the SHR group in the thalamus, dorsal thalamus, and hypothalamus (Table 2 and Figures 2(c) and 2(d)).

On days 1 and 8, glucose metabolism was higher in the SHR + LR3 group than in the SHR + sham group in the fimbria of hippocampus and caudate putamen. Glucose metabolism was lower in the SHR + LR3 group than in the SHR + sham group in the sensory cortex (Table 3 and Figures 2(e) and 2(f)).

## 4. Discussion

In the present study, we found that needling at LR3 for 10 min reduced in SHRs over a period of 60 min. This finding is consistent with other researches describing the ability of needling at LR3 to reduce (especially SBP) in hypertensive rat models and patients with hypertension [7, 17]. As acupuncture is a general treatment with “pleiotropic” responses, the simultaneous activation of multiple therapeutic mechanisms is expected [12]. Clinical acupuncture often involves repeated acupuncture treatments; indeed, repetitive acupuncture produces molecular changes and long-term cardiovascular effects that far outweigh the effects of one-time acupuncture stimulation [18]. In our study, BP was reduced for 48 h after completion of the 7-day acupuncture treatment period. Previous studies have described two possible mechanisms for the long-term cardiovascular effects of acupuncture. First, acupuncture may affect a reciprocating reinforcing circuit between the ventral hypothalamic arcuate nucleus and the midbrain ventrolateral periaqueductal gray. Second, acupuncture elicits the prolonged release of neurotransmitters and neuropeptides such as *γ*-aminobutyric acid (GABA) and opioids in the rostral ventrolateral medulla (rVLM) and other regions [18]. Therefore, while the mechanisms by which acupuncture at LR3 improves hypertension remain unclear, several studies [10] have described the antihypertensive effect and mechanism.

In this study, we used PET-CT as a novel approach to investigate the effects of needling at LR3 on various brain regions. In the SHR groups, ^18^F-FDG metabolism was lower at baseline in the medulla oblongata, cerebellum, and several cortical areas compared to the WKY group but increased after needling at LR3. It is well known that neurons are unable to synthesize or store glucose; therefore they are only dependent on glucose import. In human tissues, glucose is metabolized through glycolysis in the cytosol [19, 20]. As neurons present relatively weak expression of glycolytic enzymes, they favor another important metabolic pathway in glucose oxidation, the pentose phosphate pathway (PPP) [21]. Thus, we speculate that changes of glucose metabolism in different brain regions of rats after needling may be related to the regulation of PPP by acupuncture. Increased reactive oxygen species (ROS) formation (termed “oxidative stress”) precedes development of hypertension in SHRs, suggesting that ROS participate in the development and maintenance of hypertension [22]. Several studies have shown that the PPP is upregulated when the brain is subjected to abnormal oxidative stress, and the PPP may play a role of protecting brain against oxidative stress in neurological diseases by reducing the amounts of ROS [23]. PPP largely contributes to neuronal protection against oxidative injury by reducing NADP to NADPH [21]. Therefore, we speculate that acupuncture may reduce BP by inhibiting oxidative stress response, which is regulated by ROS formation and PPP. In our previous study, the proteomic response that contributes to the SHR phenotype and reduction of hypertension in LR3-needled rats includes the modulation of seven proteins related to oxidative stress in the medulla oblongata, including SOD, ALDH2, GSTM5, GLUD1, protein DJ-1, Hsp90a, and a-ETF [12]. The above results indicated that acupuncture may exert antihypertensive effect directly or indirectly by inhibiting oxidative stress.

Additionally, several medulla neurotransmitters are involved in cardiovascular regulation. NO is one such molecule that serves a variety of physiological functions, which is a potent vasodilator [24]. There are 3 isoforms of NO synthase (NOS): calcium-dependent endothelial NOS (eNOS), neuronal NOS (nNOS), and calcium-independent inducible NOS (iNOS). One study reported that the therapeutic effects of acupuncture in experimental renovascular hypertension were related to decreases in NOS expression. nNOS-derived NO in the rVLM induced sympathetic activation by receptor activation, whereas iNOS-derived NO induced sympathetic blockade mediated by GABA. Acupuncture reduced and downregulated nNOS protein and mRNA expression while it upregulated iNOS expression in the medulla oblongata of a stress-induced hypertensive rat model. These results suggest that acupuncture affects hypertension by activating the central sympathetic inhibitory pathway and attenuating the central sympathetic excitatory pathway through a mechanism partly involving NO [25].

It is well known that the hypothalamus plays an important role in regulation. The hypothalamus is the most advanced integration centre below the cerebral cortex which regulates the activity of the autonomic nervous system; its afferent impulses arrive from the marginal forebrain, thalamus, and brainstem reticular structures, and these efferent impulses reach these areas. The hypothalamus affects the autonomic nervous system by modulating the activity of these regions through neurotransmitter and neuroendocrine communication. A number of regions in the hypothalamus, midbrain, and medulla receive somatic input during electroacupuncture or acupuncture. Neurons in these regions are activated through a network of projections extending from the hypothalamus to more caudal regions such as the ventrolateral periaqueductal gray and rVLM through neurotransmitters including opioids, GABA, nociception, and serotonin which act postsynaptically to directly or indirectly modulate autonomic outflow [18]. Repeated needling at PC5-6 and the underlying median nerve in normotensive rats increase proenkephalin release in the rVLM. Angiotensin II (Ang II) is a key mediator in the development and the maintenance of hypertension which exerts effects on brain circulation by regulating vascular structures and two main mechanisms that control cerebral blood flow [26]. Most central actions of Ang II are mediated by activation of the Ang II type 1 receptor (AT1R). AT1Rs are expressed in the sensory circumventricular organs, hypothalamus, and brainstem in several mammals including humans [27]. Cerebral angiotensinogen, which is primarily produced by astrocytes in the thalamus, hypothalamus, and brain stem as well as cortical neurons, can convert Ang I to Ang II and elicit Ang II secretion [26]. Furthermore, studies have shown that Ang II and oxidative stress increase neuronal activity in the paraventricular nucleus and rVLM as a mechanism maintaining sympathetic activation of the cardiovascular system in patients with renovascular hypertension [28]. Moreover, the *δ*-opioid system in the rVLM partly mediates the long-term antihypertensive effects of electroacupuncture; needling at ST36-ST37 in rats with cold-induced hypertension increased the mRNA expression of preproenkephalin in the rVLM for up to 48 h after treatment [29]. These mechanisms may also be involved in the LR3-needled effect in the hypothalamus and thalamus of SHRs.

Finally, it is important to note that our research demonstrates a point-specific effect of acupuncture in hypertension by using the SHR + sham group as a nonacupuncture control. Glucose metabolism was differentially altered in the SHR + LR3 group compared to the SHR + sham group in several brain regions including the sensory cortex and thalamus. Additionally, LR3 needling produced a better antihypertensive effect than sham needling.

The present study had several limitations. We cannot directly explain the relationship between brain glucose metabolism and prognosis in hypertensive animals. Future studies should independently investigate brain regions of interest using gene chip technology in order to better inform the therapeutic mechanism of acupuncture in hypertension. Our current research focuses on the effect of acupuncture on BP and the identification of changes of glucose metabolism of target brain regions in SHRs, and the results showed that there was no antihypertensive effect at 72 hours after 7-day acupuncture. In order to study the molecular mechanism of acupuncture on the regulation in the target brain region, we sacrificed the rats quickly to perform DNA sequencing after scanning. Therefore, we cannot afford to track these rats and perform long-term longitudinal studies of acupuncture efficacy, but we are clear that it is meaningful. In the future, we will conduct further studies on the mechanism of long-term efficacy of acupuncture for the treatment of hypertension. Indeed, there are many other valuable acupoints to reduce BP, such as KI3, ST36, or acupoint compatibility [6, 9, 30], which is also one of our future research directions.

## 5. Conclusion

Using the SHR model of hypertension, we demonstrated that acupuncture at LR3 not only decreased but also altered cerebral glucose metabolism in the hypothalamus, thalamus, medulla oblongata, and cerebellum. Further investigation is needed to clarify the significance of these alterations and the mechanisms of action underlying the therapeutic effects of acupuncture in hypertension.

## Figures and Tables

**Table 1:** Sites of changed brain glucose metabolism in the SHR group versus the WKY group at 60 min after initial acupuncture or 24 h after completion of the 7-day treatment period.

Anatomical structure	Max_*T*	Peak coordinates (mm)		
		*X*	*Y*	*Z*
Day 1 (↓)				
Motor cortex	6.60	1.76	−0.04	−2.52
Sensory cortex	6.06	3.33	0.86	−0.12
Visual cortex	5.68	5.28	2.33	−5.64
Day 1 (↑)				
Infralimbic cortex	5.79	−1.27	4.78	3.72
Thalamus	7.66	−1.97	6.79	−3.00
Ventral orbital cortex	6.49	−1.68	3.96	4.44
Dorsal thalamus lateral nucleus group	7.83	−1.97	6.81	−3.24
Dorsal thalamus	7.83	−1.97	6.81	−3.24
Orbital cortex	6.49	−1.68	3.96	4.44
Hypothalamus	5.31	5.14	3.18	−5.40
Day 8 (↓)				
Cingulate cortex	6.17	1.19	2.15	0.36
Basal ganglia	7.79	1.99	2.83	−0.12
Caudate putamen	7.79	1.99	2.83	−0.12
Corpus callosum	7.62	1.86	2.65	0.12
Cingulate gyrus	6.20	1.05	2.44	0.36
Motor cortex	8.03	1.36	−0.19	−2.76
Sensory cortex	8.15	4.16	0.49	−2.04
Visual cortex	6.06	4.06	0.55	−4.44
Day 8 (↑)				
Infralimbic cortex	7.02	−0.87	4.64	3.96
Thalamus	5.01	−3.14	5.08	−5.88
Ventral orbital cortex	6.67	−1.27	4.47	3.96
Cerebellum anterior lobe	5.22	−3.76	5.03	−9.00
Dorsal thalamus lateral	5.01	−3.14	5.08	−5.88
nucleus group				
Dorsal thalamus	5.01	−3.14	5.08	−5.88
Orbital cortex	6.98	−1.00	4.63	3.96

The upward arrow (↑) indicates increased glucose metabolism, and the downward arrow (↓) indicates decreased glucose metabolism.

**Table 2:** Sites of changed brain glucose metabolism in the SHR + LR3 group versus the SHR group at 60 min after initial acupuncture or 24 h after completion of the 7-day treatment period.

Anatomical structure	Max_*T*	Peak coordinates (mm)		
		*X*	*Y*	*Z*
Day 1 (↓)				
Agranular insular cortex	4.00	−2.87	3.50	3.96
Anterior olfactory nucleus	4.90	−1.13	6.00	3.24
Accumbens nucleus	5.51	−1.39	5.57	3.00
Basal ganglia	5.24	−2.24	7.55	−3.48
Caudate putamen	4.60	−1.65	5.28	2.76
Infralimbic cortex	4.78	−1.27	4.78	3.72
Lateral orbital cortex	4.76	−1.94	3.80	4.44
Medial orbital cortex	3.44	−0.61	4.90	4.44
Medial amygdaloid nucleus	3.60	−2.93	8.24	−1.80
Olfactory bulb	4.09	−1.58	4.01	7.32
Prelimbic cortex	3.69	−1.14	4.02	4.20
Thalamus	7.46	−2.11	7.35	−2.76
Ventral orbital cortex	5.57	−1.26	5.55	3.24
Ventral pallidum	3.58	−0.98	7.09	2.52
Anterior commissure	3.90	−1.15	5.41	4.92
Amygdaloid body	3.51	−3.05	8.27	−2.28
Dorsal thalamus lateral nucleus group	7.11	−1.97	7.08	−3.00
Dorsal thalamus	7.11	−1.97	7.08	−3.00
Hypothalamus tuberal region	7.19	−2.11	7.49	−2.76
Internal capsule	5.64	−2.51	7.62	−2.76
Nucleus around the septal area	5.51	−1.39	5.57	3.00
Orbital cortex	5.21	−1.67	4.76	3.72
Day 1 (↑)				
Cerebellum anterior lobe	5.17	−3.60	5.10	−11.40
Cerebellum posterior lobe	4.47	−1.44	6.50	−12.60
Medulla oblongata	4.39	−1.72	6.88	−12.12
Sensory cortex	4.43	4.95	3.96	−1.08
Day 8 (↓)				
Lateral dorsal thalamus	3.49	2.55	5.03	−1.56
Nucleus group				
Dorsal thalamus	3.49	2.55	5.03	−1.56
Striatum	3.64	3.08	4.43	−1.08
Day 8 (↑)				
Medulla oblongata	5.51	−1.71	7.80	−12.60

The upward arrow (↑) indicates increased glucose metabolism, and the downward arrow (↓) indicates decreased glucose metabolism.

**Table 3:** Sites of changed brain glucose metabolism in the SHR + LR3 group versus the SHR + sham group at 60* *min after initial acupuncture or 24 h after completion of the 7-day treatment period.

Anatomical structure	Max_*T*	Peak coordinates (mm)		
		*X*	*Y*	*Z*
Day 1 (↑)				
Fimbria of hippocampus	3.23	−4.52	3.56	−2.76
Internal capsule	3.21	−4.52	3.54	−2.52
Day 8 (↓)				
Sensory cortex	4.85	6.14	4.23	−0.12
Day 8 (↑)				
Accumbens nucleus	6.68	1.96	7.12	2.52
Basal ganglia	6.29	1.96	7.56	2.52
Caudate putamen	5.01	2.23	7.03	2.04
Thalamus	4.51	1.80	8.70	−5.64
Ventral pallidum	6.29	1.96	7.56	−2.52

The upward arrow (↑) indicates increased glucose metabolism, and the downward arrow (↓) indicates decrease glucose metabolism.

## Data Availability

Readers can access the data underlying the findings of the study by contacting the corresponding author's email address (lai1023@163.com).
